# Supplementary material for: P-TRAP: a Panicle Trait Phenotyping tool
Source: BMC Plant Biol. 2013 Aug 29;13:122. doi: 10.1186/1471-2229-13-122 (PMC3848748; doi:10.1186/1471-2229-13-122)
Supplement: Additional file 7 — P-TRAP source code. The Netbeans project that contains the source code of the application. [file 1471-2229-13-122-S7.zip › javadoc.zip!/javadoc/org-basmati-workspace/org/basmati/workspace/ParticleScene.html]

ParticleScene (WorkSpace)


JavaScript is disabled on your browser.


- Package
- Class
- Tree
- Deprecated
- Index
- Help

*org.basmati.workspace 1.0*

- Prev Class
- Next Class

- Frames
- No Frames

- All Classes

- Summary:
- Nested |
- Field |
- Constr |
- Method

- Detail:
- Field |
- Constr |
- Method


org.basmati.workspace

## Class ParticleScene

- java.lang.Object
- - org.netbeans.api.visual.widget.Widget
  - - org.netbeans.api.visual.widget.Scene
    - - org.netbeans.api.visual.model.ObjectScene
      - - org.netbeans.api.visual.graph.GraphScene<org.biolag.particleprocessor.Particle,java.lang.String>
        - - org.basmati.workspace.ParticleScene

- All Implemented Interfaces:
  :   javax.accessibility.Accessible, org.openide.util.Lookup.Provider

  ---

    

  ```
  public class ParticleScene
  extends org.netbeans.api.visual.graph.GraphScene<org.biolag.particleprocessor.Particle,java.lang.String>
  ```

  The `GraphScene` which shows the grains and allows the user to edit
  them.

  Author:
  :   faroq

- - ### Nested Class Summary

    - ### Nested classes/interfaces inherited from class org.netbeans.api.visual.graph.GraphScene

      `org.netbeans.api.visual.graph.GraphScene.StringGraph`
    - ### Nested classes/interfaces inherited from class org.netbeans.api.visual.widget.Scene

      `org.netbeans.api.visual.widget.Scene.SceneListener`
    - ### Nested classes/interfaces inherited from class org.netbeans.api.visual.widget.Widget

      `org.netbeans.api.visual.widget.Widget.Dependency`
  - ### Constructor Summary

    Constructors

    Constructor and Description

    `ParticleScene()`
  - ### Method Summary

    Methods

    | Modifier and Type | Method and Description |
    | --- | --- |
    | `protected void` | `attachEdgeSourceAnchor(java.lang.String e, org.biolag.particleprocessor.Particle n, org.biolag.particleprocessor.Particle n1)` |
    | `protected void` | `attachEdgeTargetAnchor(java.lang.String e, org.biolag.particleprocessor.Particle n, org.biolag.particleprocessor.Particle n1)` |
    | `protected org.netbeans.api.visual.widget.Widget` | `attachEdgeWidget(java.lang.String e)` |
    | `protected org.netbeans.api.visual.widget.Widget` | `attachNodeWidget(org.biolag.particleprocessor.Particle particle)` |
    | `boolean` | `checkResults()` |
    | `void` | `clear()` |
    | `void` | `fit2view(javax.swing.JScrollPane scrollpane)` |
    | `java.io.File` | `getImagefile()` |
    | `org.biolag.particleprocessor.ParticlesModel` | `getModel()` |
    | `java.awt.Image` | `getOriginalImage()` |
    | `org.biolag.particleprocessor.ParticlesModel` | `getUpdatedParticles()` |
    | `boolean` | `isDirty()` |
    | `void` | `setDirty(boolean dirty)` |
    | `void` | `setImagefile(java.io.File imagefile)` |
    | `void` | `setModel(org.biolag.particleprocessor.ParticlesModel model)` |
    | `void` | `setOriginalImage(java.awt.Image originalImage)` |
    | `void` | `setupImageWidget()` |
    | `void` | `setuplayers()` |
    | `void` | `setUpmodel()` |
    | `org.biolag.particleprocessor.Particle` | `swapXY(org.biolag.particleprocessor.Particle particle)` |
    | `void` | `updateImagewidget()` |
    | `void` | `updateParticlesModel(boolean swapXY)` Update the particles model in order to save it to a file |

    - ### Methods inherited from class org.netbeans.api.visual.graph.GraphScene

      `addEdge, addNode, detachEdgeWidget, detachNodeWidget, findEdgesBetween, findNodeEdges, getEdges, getEdgeSource, getEdgeTarget, getNodes, isEdge, isNode, notifyEdgeAdded, notifyNodeAdded, removeEdge, removeNode, removeNodeWithEdges, setEdgeSource, setEdgeTarget`
    - ### Methods inherited from class org.netbeans.api.visual.model.ObjectScene

      `addObject, addObjectSceneListener, createObjectHoverAction, createSelectAction, findObject, findStoredObject, findWidget, findWidgets, getFocusedObject, getHighlightedObjects, getHoveredObject, getIdentityCode, getObjects, getObjectState, getSelectedObjects, isObject, removeObject, removeObjectSceneListener, setFocusedObject, setHighlightedObjects, setHoveredObject, setSelectedObjects, userSelectionSuggested`
    - ### Methods inherited from class org.netbeans.api.visual.widget.Scene

      `addSceneListener, convertSceneToView, convertSceneToView, convertViewToScene, createBirdView, createSatelliteView, createView, createWidgetHoverAction, getActiveTool, getDefaultFont, getFocusedWidget, getGraphics, getInputBindings, getKeyEventProcessingType, getLookFeel, getMaximumBounds, getPriorActions, getResourceTable, getSceneAnimator, getView, getZoomFactor, isRepaintRequiredForRevalidating, isValidated, paint, removeSceneListener, setActiveTool, setFocusedWidget, setKeyEventProcessingType, setLookFeel, setMaximumBounds, setResourceTable, setZoomFactor, validate, validate`
    - ### Methods inherited from class org.netbeans.api.visual.widget.Widget

      `addChild, addChild, addChild, addChild, addChildren, addDependency, bringToBack, bringToFront, calculateClientArea, convertLocalToScene, convertLocalToScene, convertSceneToLocal, convertSceneToLocal, createActions, equals, getAccessibleContext, getActions, getActions, getBackground, getBorder, getBounds, getChildConstraint, getChildren, getClientArea, getCursor, getCursorAt, getDependencies, getFont, getForeground, getLayout, getLocation, getLookup, getMaximumSize, getMinimumSize, getParentWidget, getPreferredBounds, getPreferredLocation, getPreferredSize, getScene, getState, getToolTipText, hashCode, isCheckClipping, isEnabled, isHitAt, isOpaque, isPreferredBoundsSet, isVisible, notifyAdded, notifyBackgroundChanged, notifyFontChanged, notifyForegroundChanged, notifyRemoved, notifyStateChanged, paint, paintBackground, paintBorder, paintChildren, paintWidget, removeChild, removeChildren, removeChildren, removeDependency, removeFromParent, repaint, resolveBounds, revalidate, revalidate, setAccessibleContext, setBackground, setBackgroundFromResource, setBorder, setBorder, setCheckClipping, setChildConstraint, setCursor, setEnabled, setFont, setFontFromResource, setForeground, setForegroundFromResource, setLayout, setMaximumSize, setMinimumSize, setOpaque, setPreferredBounds, setPreferredLocation, setPreferredSize, setState, setToolTipText, setVisible, updateResources`
    - ### Methods inherited from class java.lang.Object

      `clone, finalize, getClass, notify, notifyAll, toString, wait, wait, wait`

- - ### Constructor Detail


    - #### ParticleScene

      ```
      public ParticleScene()
      ```
  - ### Method Detail


    - #### isDirty

      ```
      public boolean isDirty()
      ```


    - #### setDirty

      ```
      public void setDirty(boolean dirty)
      ```


    - #### attachNodeWidget

      ```
      protected org.netbeans.api.visual.widget.Widget attachNodeWidget(org.biolag.particleprocessor.Particle particle)
      ```

      **Specified by:**
      :   `attachNodeWidget` in class `org.netbeans.api.visual.graph.GraphScene<org.biolag.particleprocessor.Particle,java.lang.String>`


    - #### getImagefile

      ```
      public java.io.File getImagefile()
      ```


    - #### setImagefile

      ```
      public void setImagefile(java.io.File imagefile)
      ```


    - #### getModel

      ```
      public org.biolag.particleprocessor.ParticlesModel getModel()
      ```


    - #### setModel

      ```
      public void setModel(org.biolag.particleprocessor.ParticlesModel model)
      ```


    - #### setuplayers

      ```
      public void setuplayers()
      ```


    - #### setupImageWidget

      ```
      public void setupImageWidget()
      ```


    - #### getOriginalImage

      ```
      public java.awt.Image getOriginalImage()
      ```


    - #### setOriginalImage

      ```
      public void setOriginalImage(java.awt.Image originalImage)
      ```


    - #### updateImagewidget

      ```
      public void updateImagewidget()
      ```


    - #### setUpmodel

      ```
      public void setUpmodel()
      ```


    - #### updateParticlesModel

      ```
      public void updateParticlesModel(boolean swapXY)
      ```

      Update the particles model in order to save it to a file


    - #### getUpdatedParticles

      ```
      public org.biolag.particleprocessor.ParticlesModel getUpdatedParticles()
      ```


    - #### swapXY

      ```
      public org.biolag.particleprocessor.Particle swapXY(org.biolag.particleprocessor.Particle particle)
      ```


    - #### checkResults

      ```
      public boolean checkResults()
      ```


    - #### clear

      ```
      public void clear()
      ```


    - #### fit2view

      ```
      public void fit2view(javax.swing.JScrollPane scrollpane)
      ```


    - #### attachEdgeWidget

      ```
      protected org.netbeans.api.visual.widget.Widget attachEdgeWidget(java.lang.String e)
      ```

      **Specified by:**
      :   `attachEdgeWidget` in class `org.netbeans.api.visual.graph.GraphScene<org.biolag.particleprocessor.Particle,java.lang.String>`


    - #### attachEdgeSourceAnchor

      ```
      protected void attachEdgeSourceAnchor(java.lang.String e,
                                org.biolag.particleprocessor.Particle n,
                                org.biolag.particleprocessor.Particle n1)
      ```

      **Specified by:**
      :   `attachEdgeSourceAnchor` in class `org.netbeans.api.visual.graph.GraphScene<org.biolag.particleprocessor.Particle,java.lang.String>`


    - #### attachEdgeTargetAnchor

      ```
      protected void attachEdgeTargetAnchor(java.lang.String e,
                                org.biolag.particleprocessor.Particle n,
                                org.biolag.particleprocessor.Particle n1)
      ```

      **Specified by:**
      :   `attachEdgeTargetAnchor` in class `org.netbeans.api.visual.graph.GraphScene<org.biolag.particleprocessor.Particle,java.lang.String>`


- Package
- Class
- Tree
- Deprecated
- Index
- Help

*org.basmati.workspace 1.0*

- Prev Class
- Next Class

- Frames
- No Frames

- All Classes

- Summary:
- Nested |
- Field |
- Constr |
- Method

- Detail:
- Field |
- Constr |
- Method
